# Skateboards: Are they really perilous? A retrospective study from a district hospital

**DOI:** 10.1186/1756-0500-1-59

**Published:** 2008-07-31

**Authors:** Ulfin Rethnam, Rajam Sheeja Yesupalan, Amit Sinha

**Affiliations:** 1 Department of Orthopaedics, Glan Clwyd Hospital, Bodelwyddan, UK; 2 Department of Accident & Emergency, Glan Clwyd Hospital, Bodelwyddan, UK; 3 Department of Orthopaedics, Glan Clwyd Hospital, Bodelwyddan, UK

## Abstract

**Background:**

Skateboarding has been a popular sport among teenagers even with its attendant associated risks. The literature is packed with articles regarding the perils of skateboards. Is the skateboard as dangerous as has been portrayed?

**Methods:**

This was a retrospective study conducted over a 5 year period. All skateboard related injuries seen in the Orthopaedic unit were identified and data collated on patient demographics, mechanism & location of injury, annual incidence, type of injury, treatment needed including hospitalisation.

**Results:**

We encountered 50 patients with skateboard related injuries. Most patients were males and under the age of 15. The annual incidence has remained low at about 10. The upper limb was predominantly involved with most injuries being fractures. Most injuries occurred during summer. The commonest treatment modality was plaster immobilisation. The distal radius was the commonest bone to be fractured. There were no head & neck injuries, open fractures or injuries requiring surgical intervention.

**Conclusion:**

Despite its negative image among the medical fraternity, the skateboard does not appear to be a dangerous sport with a low incidence and injuries encountered being not severe. Skateboarding should be restricted to supervised skateboard parks and skateboarders should wear protective gear. These measures would reduce the number of skateboarders injured in motor vehicle collisions, reduce the personal injuries among skateboarders, and reduce the number of pedestrians injured in collisions with skateboarders.

## Findings

Skateboarding is a popular recreational activity among youngsters. The capability of attaining speeds up to 40mph and the possibility of performing various tricks have added a thrill factor to this sport [1]. Its inherent instability adds to the excitement of skateboarding. There is little wonder that a wide range of injuries are seen with skateboarding.

With reported deaths and an ever increasing morbidity, there have been calls to "ban the boards" [2]. Most studies highlight the dangers of skateboarding [3-5]. There are other studies that suggest that most skateboard injuries are minor [6,7].

Is the skateboard really dangerous? Is the call to ban skateboards justifiable? We aimed to answer these questions in our study.

## Methods

This was a retrospective analysis of skateboard injuries encountered by the Orthopaedic unit in a busy district hospital that caters to a population of about 100000, 28% being children. Being a popular holiday haven, the population triples during the summer. The Orthopaedic unit receives about 2200 trauma admissions, performs 1700 trauma procedures and assesses 5000 new patients in the fracture clinics annually.

Over a five year period (2002 – 2006) we included all skateboard related injuries seen by the Orthopaedic unit. Data was obtained from patient records and radiographs. The following data was collected:

1. Patient demographics

2. Annual incidence of skateboard related injuries

3. Mechanism of injury

4. Location where injury occurred

5. Seasonal variation

6. Injuries seen

7. Hospitalisations following skateboard related injuries

8. Surgical interventions

9. Treatment

10. Deaths

## Results

**Figure 1:**
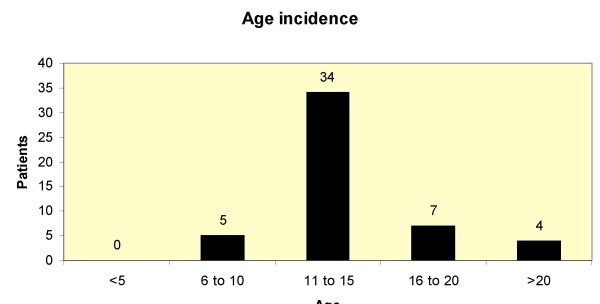
Age incidence of skateboard injuries.

We encountered 50 patients with skateboard related injuries during the study period. (Table 1) of whom 40 were males and 39/50 were <15 years of age. The mean age was 15.3 years (Range 6 – 50). Patients were divided into 5 age groups: 0–5, 6–10, 11–15, 16–19, >20 years. (Figure 1)

**Table 1:** Patient profile, annual incidence, injuries & treatment

Patient ID	Age	Sex	Year	Diagnosis	Treatment	Hospitalisation
1	15	M	2002	Fracture distal radius	Plaster	No
2	13	F	2002	Soft tissue injury wrist	Splint	No
3	11	M	2002	Fracture proximal phalanx middle finger	Neighbour strapping	No
4	11	M	2002	Fracture medial cuneiform	Plaster	Yes
5	13	M	2002	Fracture distal tibia	Plaster	Yes
6	16	M	2002	Fracture 4th metacarpal	Plaster	No
7	12	M	2003	Fracture proximal phalanx thumb	Thumb spica plaster	No
8	11	M	2003	Fracture clavicle	Sling	No
9	12	M	2003	Fracture femur	Traction	Yes
10	15	M	2003	Fracture index finger middle phalanx	Neighbour strapping	No
11	16	M	2003	Fracture ring finger proximal phalanx	Neighbour strapping	No
12	11	M	2003	Fracture calcaneum	Plaster	No
13	40	M	2003	Fracture radial head	Sling	No
14	12	M	2003	Fracture proximal phalanx thumb	Thumb spica plaster	No
15	18	M	2003	Fracture greater tuberosity humerus	Sling	No
16	10	F	2003	Soft tissue injury wrist	Plaster	No
17	12	M	2003	Fracture third metacarpal neck	Plaster	No
18	15	M	2003	Fracture second metacarpal neck	Plaster	No
19	11	M	2004	Soft tissue injury wrist	Plaster	No
20	17	M	2004	Ankle sprain	Tubigrip	No
21	16	M	2004	Fracture distal radius	Plaster	Yes
22	11	F	2004	Soft tissue injury shoulder	Sling	No
23	18	M	2004	Fracture ulnar styloid	Plaster	No
24	16	M	2004	Fracture radial head	Sling	No
25	6	F	2004	Fracture distal radius	Plaster	No
26	11	M	2004	Soft tissue injury knee	Tubigrip	No
27	14	M	2004	Soft tissue injury wrist	Plaster	No
28	14	M	2005	Fracture lateral malleolus	Plaster	No
29	13	F	2005	Fracture lateral malleolus	Plaster	No
30	13	F	2005	Fracture lateral malleolus	Plaster	No
31	12	M	2005	Fracture distal radius	Plaster	No
32	11	M	2005	Fracture ring finger proximal phalanx	Neighbour strapping	No
33	15	M	2005	Soft tissue injury wrist	Splint	No
34	50	M	2005	Fracture distal radius	Plaster	No
35	50	M	2005	Fracture radial head	Plaster	No
36	12	M	2005	Fracture distal radius and ulna	Plaster	Yes
37	11	F	2006	Fracture distal radius	Plaster	No
38	10	M	2006	Lateral collateral ligament injury knee	Splint	No
39	14	F	2006	Bimalleolar fracture ankle	Plaster	No
40	14	F	2006	Bimalleolar fracture ankle	Plaster	Yes
41	10	M	2006	Soft tissue injury knee	Splint	No
42	6	F	2006	Fracture clavicle	Sling	No
43	13	M	2006	Fracture distal radius	Plaster	No
44	14	M	2006	Dislocation DIPJ little finger	Neighbour strapping	No
45	33	M	2006	Fracture radial head	Sling	No
46	14	M	2006	Fracture fifth metacarpal neck	Plaster	No
47	11	M	2006	Fracture distal radius	Plaster	No
48	14	M	2006	Fracture distal radius	Plaster	Yes
49	13	M	2006	Fracture 2–5 metacarpal necks	Plaster	No
50	15	M	2006	Soft tissue injury wrist	Splint	No

### Annual incidence and seasonal trend

**Figure 2:**
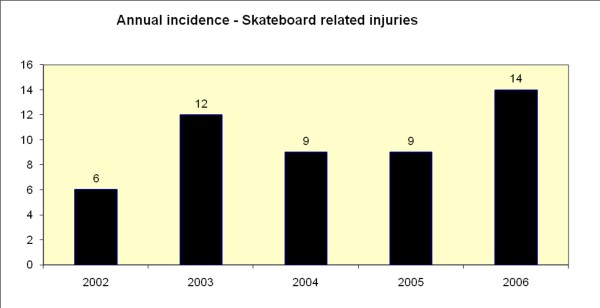
Annual incidence of skateboard related injuries encountered.

The annual incidence did not vary to a large extent. (Figure 2) The mean incidence was 10 patients per year (Range 6 – 14). Most injuries were sustained during the summer (36/50).

### Mechanism and location of injuries

Most injuries (28/50) occurred while performing a trick on the skateboard, 1 patient was involved in a motor vehicle collision. The remaining patients lost balance and fell while on the skateboard. (Table 2)

**Table 2:** Mechanism of injury & location where injury sustained

Injury mechanism	
Vehicle Collision	1
During trick	28
Lost balance	21

Location	

Road	1
Skateboard park	21
Pavement	28

In 28/50 patients, the injury event occurred on the pavement. The rest of the injuries were sustained in a skateboard park or on the road. (Table 2)

### Type of injury

Most injuries affected the upper limbs (37/50) with 39/50 being fractures.

Among the upper limb injuries seen, 30/50 were fractures while the rest were soft tissue injuries. Fractures were common in the hand (12/37), while the commonest upper limb region affected was the wrist (16/37). The distal radius was the most common upper limb fracture. (10/30) (Table 3)

**Table 3:** Upper limb injuries encountered

Fracture phalanges	6
Fracture metacarpals	5
Fracture radius & ulna	1
Fracture distal radius	9
Fracture ulnar styloid	1
Fracture radial head	4
Fracture clavicle	2
Fracture proximal humerus	1
Dislocation DIPJ little finger	1
Soft tissue injury wrist	6
Soft tissue injury shoulder	1

In the lower limb, 9/13 injuries were fractures. The ankle was the most injured region (7/13), with fractures around the ankle being the most common lower limb fracture (5/13). (Table 4)

**Table 4:** Lower limb injuries encountered

Fracture lateral malleolus	3
Bimalleolar fracture ankle	2
Fracture calcaneum	1
Fracture medial cuneiform	1
Fracture distal tibia	1
Fracture femur	1
Ankle sprain	1
Lateral collateral ligament sprain knee	1
Soft tissue injury knee	2

The most serious injury encountered was a femoral fracture that required traction.

### Treatment

**Figure 3:**
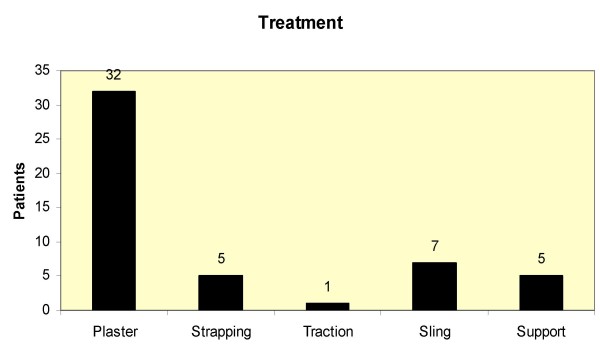
Treatment modalities for skateboard injuries encountered.

The most common treatment modality was immobilisation in plaster (32/50). (Figure 3) Manipulation and reduction of fractures/dislocations were needed in 2/50 patients. None of the injuries required surgical intervention and only 7/50 patients required hospitalisation for observation.

## Discussion

Skateboarding as a recreational sport has been around since the 1960s. Since its introduction design changes and improvement in the manufacturing materials, especially the poly-urethane wheels, has made the skateboard more manoeuvrable. The skateboard can reach up to speeds of 40 mph and a variety of tricks can be performed on them [1]. There has therefore been a rise in its popularity amongst youngsters.

Papers on skateboard injuries have been published since the 1960s [7]. With the rising incidence of injuries, the skateboard was even referred to as a "medical menace" [4]. The calls to ban the skateboard resulted in banning this popular sport from public roads and sidewalks in Sweden. In Norway, there was a complete ban on skateboarding in the 1980s [4].

Some articles use terms like "hazard" & "perilous" when describing the skateboard. Most articles highlight the various injuries sustained while skateboarding [3,4,7-9]. The skateboard has been portrayed as a villain causing increasing morbidity among youngsters.

Our department caters to a population of about 100,000, 28% being children. Skateboarding is a popular activity amongst youngsters in this region. There are a few skateboard parks in our locality. This study was initiated as the skateboard injuries seen by us were not severe and the numbers were insignificant. Our question was whether the skateboard deserved all the negative publicity among the medical community, it being popular among youngsters.

The incidence of skateboard injuries reported in the literature is varied [10,11]. Zalvaras et al found only 187 skateboard injuries among 2371 fractures seen in a level1 trauma centre paediatric fracture clinic [12]. In a study by Schalamon J et al, the 4-month calculated incidence in children less than 16 years of age was 0.68 per 1,000 for skateboard injuries [7]. They suggested that skateboard injuries accounted for 2.6% of all paediatric traumas within a region. Our annual incidence was 10 patients per year among 5000 new fracture clinic attendances (2 per 1000). Compared to other recreational activities like scooter riding, roller skating and in-line skating, the incidence of skateboard related injuries is varied [7,11,12]. The injury characteristics seem to be similar among these activities with forearm injuries being more common [11]. The reported severity of injuries related to skateboards compared to the other activities has been varied in the literature [2,11].

Head and neck injuries following skateboard accidents are commonly seen in children younger than 5 years [7]. The incidence of head injuries and critical injuries due to skateboarding accidents has been reported to be high [3]. We did not encounter any open fractures or head and neck injuries relating to skateboard injuries. Most skateboarding injuries reported in the literature are minor although the occurrence of potentially life threatening injuries has been documented [4].

In our study although most injuries were fractures, these were not severe and were managed conservatively. The most severe injury was a femoral fracture that was managed successfully in traction. Few patients (4%) required a manipulation for their fractures and only 14% needed to be hospitalised. This correlates with other studies in the literature. Illingworth et al encountered 40.9% fractures in 225 skateboard injuries of which only 19 patients required a manipulation under anaesthesia [8]. Our study did show that most injuries occurred on the pavement. To enhance safety while skateboarding, we agree with other studies with regards to encouraging youngsters to use supervised skateboard parks and use of protective gear [7,10].

Our study has its limitations in that only patients encountered by the Orthopaedic unit were included. Patients with minor injuries like contusions & sprains may have been discharged from the Accident & Emergency. This would still show that most injuries with skateboard accidents are minor. We did not look at the effect of use of protective gear on skateboard injuries which is a limitation of the study. Based on the results of our study, the skateboard is not a dangerous sport and calls to ban this popular sport is not justified.

## Conclusion

Our study found that skateboarding injuries, although present, were infrequent and not severe to call for banning of the sport. Use of protective gear and skateboard parks may lower the risk of injuries.

## Competing interests

The authors declare that they have no competing interests.

## Authors' contributions

UR, the main author was responsible for conducting the study, acquisition, analysis and interpretation of the data and preparing the manuscript.

RSY, the co-author was responsible for literature review, data acquisition and has approved the final draft.

AS, the senior author was responsible for supervising the study, proof reading of the manuscript and has approved the final draft of the manuscript.
